# Undertaking Research in Other Countries: Author's Reply

**DOI:** 10.1371/journal.pmed.0040188

**Published:** 2007-05-29

**Authors:** Loane Skene

Adamson Muula [1] rightly observes that demarcation between the “zones” of Skene's barometer is unclear. Certain activities may fall in one zone or another, depending on the circumstances. A research project does not fall within a particular zone solely because of its type. One must consider the project in operation. Muula mentions the treatment of participants in trials, particularly whether they are properly informed before the trial starts. One could add other factors such as the way participants are recruited, personal information held, or adverse incidents reported. Thus “research involving competent adults”, which I have in the green zone (permitted with ethical oversight), would move to the yellow or orange zones (permitted under national laws with ethical oversight; or prohibited by national laws) if participants were coerced or duped into entering a trial; or if their personal details were revealed without their consent or other lawful authority; or if researchers did not inform the appropriate authority during the trial that other participants had suffered serious and unexpected injury. Similarly, “medical research involving children” would fall within the green zone only if there is minimal harm to the child and full parental consent.

I suggested in my original article that the sensitivity of research could be “measured” on the barometer by its nature and by how it is legally regulated. I then gave examples, such as medical research involving competent adults and research on genetically modified crops. That is, of course, only the first step. I intended to designate research of that kind that is “properly conducted”. The zones are less clear when one considers a variation of the initial research activity.

This is illustrated by Muula's examples. In Australia, there is no general legal restriction on observing people in a public place, or even photographing or videotaping them for research. I therefore placed those activities in the white zone on the Australian barometer (no specific laws or oversight). Australian law does not recognize a general legal right to “privacy”. However, those activities could appear in another zone in certain circumstances. It is an offence to “stalk” people, or to photograph them so as to suggest they are acting unlawfully or to defame them.

Similarly, in Australia, it is not unlawful to collect human bodily material that has been “discarded” by other people, even if they have not consented and do not know about it. A scientist who picks up a tissue with a person's blood on it, in a public place, and does research on the blood would commit no offence even if he or she knew the identity of the person concerned. No ethics committee approval is needed. The scientist would breach the law only if personal information derived from the research was revealed without lawful authority. The same argument applies to research on bodily materials that are discarded by hospitals or pathology laboratories as garbage. The position of the research on the barometer moves according to the circumstances. If the bodily material is specifically sought from a hospital or laboratory, ethics approval would be needed, and possibly also consent from the people concerned, though that can be waived in certain circumstances.

The barometer is always based on local laws, which are a measure of the sensitivity of the community regarding particular conduct. The barometer reading may indicate that community opinion is divided or not so opposed to particular activities that they could not be accepted in another country, especially where they are undertaken with appropriate ethical surveillance. If other countries choose to use the barometer, they must substitute their own local laws and they may, of course, reach a different conclusion. Research ethics are not universal, but there may be more agreement than is commonly thought.
